# Where Shared Sanitation is the Only Immediate Option: A Research Agenda for Shared Sanitation in Densely Populated Low-Income Urban Settings

**DOI:** 10.4269/ajtmh.20-0985

**Published:** 2020-11-16

**Authors:** James B. Tidwell, Jenala Chipungu, Ian Ross, Prince Antwi-Agyei, Mahbub-Ul Alam, Innocent K. Tumwebaze, Guy Norman, Oliver Cumming, Sheillah Simiyu

**Affiliations:** 1 World Vision Inc., Washington, District of Columbia;; 2 Harvard Kennedy School of Government, Cambridge, Massachusetts;; 3 Centre for Infectious Disease Research in Zambia, Lusaka, Zambia;; 4 London School of Hygiene and Tropical Medicine, London, United Kingdom;; 5 NHance Development Partners Ltd, Kumasi, Ghana;; 6 University of Energy and Natural Resources, Sunyani, Ghana;; 7 International Centre for Diarrhoeal Disease Research in Bangladesh, Dhaka, Bangladesh;; 8 Temple University, Philadelphia, Pennsylvania;; 9 Urban Research, Guildford, United Kingdom;; 10 African Population and Health Research Center, Nairobi, Kenya

## Abstract

Shared sanitation is not currently accepted within the international normative definitions of “basic” or “safely managed” sanitation. We argue that pro-poor government strategies and investment plans must include high-quality shared sanitation as an intermediate step in some densely populated urban areas. User experience must be considered in establishing the definition of high quality. We call for additional research on effective interventions to reach these quality standards and for the development of rigorous measures applicable to global monitoring.

## INTRODUCTION

Sustainable Development Goal (SDG) target 6.2 calls for universal access to safely managed sanitation by 2030, defined as “the use of an improved sanitation facility which is not shared with other households and where excreta is safely disposed in situ or transported and treated off-site.” Meeting this target in a rapidly urbanizing world with densifying urban spaces^1^ is profoundly challenging. Globally, 24% of urban dwellers live in informal settlements,^2^ characterized by poor housing quality, infrastructure, and services along with high population density,^3^ leading to poorer health outcomes than rural or formal urban areas.^4^ Residents in these contexts are a major contributor to the large and growing reality of shared sanitation use globally, with the number of users increasing from 249 million in 1990 to 603 million in 2015.^5^

Sanitation shared by more than one household is not considered to meet the SDG standard of either basic or safely managed sanitation under current WHO/UNICEF Joint Monitoring Programme for Water and Sanitation definitions.^6^ Shared sanitation comprises a range of technologies and management/user models, ranging from toilets shared by a small number of neighboring households to public toilets used on occasion by thousands of people. These facilities may be categorized by location, physical or social aspects of access, size, and models of ownership, management, or payment.^7^ Acceptability varies greatly across these many types of shared sanitation, although here we restrict our focus to shared household sanitation (i.e., not public toilets).

A systematic review in 2014 did not support the inclusion of shared sanitation in general as “improved” under the Millennium Development Goals,^8^ although it concluded that existing evidence was limited and of poor quality. A subsequent multi-country study with focus on moderate to severe diarrhea in children found that shared sanitation use was generally associated with higher disease risk but found it to be protective in some settings.^9^ Other recent studies have reported that toilets shared exclusively between neighbors are more protective against diarrhea than public/communal toilets.^10^ Shared toilets may also be of higher structural quality than private toilets^11^ and have similar levels of fecal contamination,^12^ although they may be less clean because more households share them.^13^ However, in India, some professionally managed public toilet blocks were found to be acceptable to users, and effluent was safely managed.^14^ Improving the quality of shared sanitation alone in high-density urban settings may only have a limited impact on health, given the pervasive contamination.^15^ Given that the use of these shared facilities was high, there is no reason to assume that non-shared, household-level facilities would have had a greater health impact, and poor waste management,^16^ child feces,^17^ and animal feces^18^ mean that many other sources of contamination are present.

In 2017, a call for further consideration of shared sanitation under the SDGs argued that ignoring the possibility of acceptable shared sanitation might limit investment in informal urban settlements.^19^ Some city sanitation master plans specifically mentioned the inclusion of shared, on-site sanitation because of its meeting the proposed SDG indicators at the time.^20^ The SDGs themselves called for intermediate steps and reducing inequalities, advocating for “prioritizing investments in high-quality shared toilets where it is the only viable option for improving sanitation services.”^19^ As new interdisciplinary research emerges on the topic of shared sanitation in low-income unplanned urban areas, we convened a symposium at the 2019 University of North Carolina Water and Health Conference to map out a potential research agenda.

Although private household sanitation remains the normative global standard and the preferred option for most users, in some settings, shared sanitation exists as a short- to medium-term necessity.^21^ The aim of this symposium was to set out a research agenda that would support effective policy and practice on shared sanitation in urban settings where this is currently considered the only option. We considered three aspects: how users experience shared sanitation, how we can improve its quality, and how we can define and measure high-quality sanitation.

### Setting and user experiences.

In most low-income areas, there is often little space for private household sanitation facilities, whether inside the dwelling or outside. Shared household sanitation services in low-income settlements are often on-site facilities provided by landowners, some of whom reside within the settlements.^22,23^ Landowners usually prioritize construction of rental units over sanitation facilities, thinking they are the better investment, thus forcing tenants to continue sharing the few available sanitation facilities. Insecurity of tenure in some settlements also contributes to substandard housing and sanitation facilities.^23,24^ When toilets are not provided or are of low quality, residents sometimes also access pay-per-use public toilets and may use many different sanitation facilities regularly even if they are provided a high-quality toilet at home.^25^ However, factors such as distance, cleanliness, cost, and operating hours influence the use of public toilet facilities.^10,26,27^ In addition, the lack of access or access to low-quality sanitation facilities also poses a greater challenge to vulnerable populations, such as women, the elderly, people with disabilities, and children.^28,29^

In rented accommodation, landlords usually have responsibility for sanitation provision, whereas tenants take responsibility for cleaning.^24^ Operation and maintenance costs and responsibilities are sometimes taken up entirely by landlords or shared between landlords and tenants. Shared sanitation facilities are often dirty because of limited participation in cleaning, especially when there are many users,^13,30,31^ experiencing the same challenges as management of any common-pool resources whose maintenance depends on actions of other users.^31,32^ The uncleanliness and lack of participation in cleaning lead to dissatisfaction among the users of shared sanitation facilities,^33^ disagreements and conflicts among users,^31,32^ and psychological stress,^34^ and in extreme cases, users can opt to use alternatives such as open defecation or “flying toilets.”^35^ User commitment and social capital are critical for successful collective management of shared sanitation facilities, including the need to strengthen communication and accountability between landlords and tenants.^32,36–38^ Our suggested priority research questions for the setting and user experience are as follows:

1. What kinds of experiences do users, and especially women, children, and those with disabilities, have with different kinds of shared sanitation?
2. What are the key drivers of demand for improving the quality of shared sanitation?
3. How can we assess the impact of shared sanitation quality on user experience?
4. What is the impact of improvements in the structural quality and management systems on the overall physical and mental well-being of shared sanitation users?

### Effective interventions.

Shared sanitation interventions generally target one or both of two key results: the cleanliness of facilities and improving structural quality through new construction or modification of existing facilities. Shared cleaning has been promoted through direct-to-household behavior change communication focused on flushing toilets, solid waste disposal, and use of a potty to collect child feces, along with the provision of water pouring cups and storage containers.^39^ Beyond this individual focus, other interventions have addressed social dilemmas and collective action problems around shared cleaning. In Uganda, meetings were held between landlords and tenants to discuss challenges and make commitments to cleaning by creating a sense of cleaning obligation, ease, approval, and changing affective beliefs.^40^ In Zambia, because of the challenges of high turnover of tenants and the existing system of verbal, daily rota turns being hard to manage, a new system was introduced to improve cleaning.^41^ A weekly rota system was promoted with a “symbol of responsibility” hanging above the door of the responsible tenant so that they were accountable for any failure to clean, resolving the key management challenge and signaling social norms to new residents without the need for whole-of-plot meetings.

Infrastructure improvement has been attempted through assessing tenant willingness to pay for better sanitation through increased rent^41^ and then leveraging that via emotional demonstrations and games to help landlords decide to intentionally improve sanitation quality.^41^ Although this approach was successful, potentially because in the study area, 42% of landlords initially thought that tenants were not willing to pay for anything beyond a basic sanitation service, communicating latent demand may only be the first step in the process of improving infrastructure quality. Some gains have also been observed by providing loans^42^ or subsidies,^43,44^ promoting regulatory enforcement^45^ and legal approaches^46^ to solving tenure challenges and through coproduction and collective action among residents.^47^ We suggest the following priority research questions on designing effective interventions:

5. What are the most effective management systems for different kinds of shared sanitation?
6. What combination and/or sequencing of market-based approaches, financial products, regulatory and legal frameworks, and collective action/coproduction is needed to drive sustainable improvements in shared sanitation quality?

### Measurement and monitoring.

Measurement of different aspects of shared sanitation quality is important for routine monitoring by service providers to global monitoring by the WHO/UNICEF Joint Monitoring Programme for Water and Sanitation to rigorous impact evaluation. Using a sanitation quality index and toilet cleanliness index, a study of Quality Indicators for Shared Sanitation in three countries found that reliance on improved technology type and toilet sharing may not serve as an adequate indicator of toilet cleanliness or overall toilet quality. Preliminary findings from this study show that in addition to toilet technology and sharing, other factors such as the location of the toilet, whether the door has a lock and whether the floor is tiled, significantly influence overall toilet quality.^48^ Characteristics of sanitation infrastructure can be objectively and rapidly measured, and structural quality had more of an impact on observed cleanliness and reported satisfaction than cleaning behavior in a study in Zambia.^49^ However, measuring infrastructure alone is insufficient. Two users, for example, an adolescent girl and a middle-aged man, may experience the same toilet very differently. Assessment of user experience and quality-of-life impacts may be required in many cases. Work is ongoing in urban Mozambique to develop a measure of sanitation-related quality of life, which captures the extent of achievement of outcomes most valued by users.^50^ For rural areas, Caruso et al.^51^ developed an experiential measure of women’s sanitation insecurity, conceptualized as an exposure, rather than an outcome. It was subsequently used to show that latrine access was associated with higher mental well-being.^52^

Those who fund, design, monitor, and evaluate programs require simple validated measures that can be applicable across settings, whether for infrastructure quality or user-reported outcomes. Many measures mentioned earlier have been developed and/or applied in only one setting thus far and insufficiently consider outcomes further down the service chain. We suggest the following priority research questions around measurement and monitoring:

7. What easy-to-use measures of shared sanitation quality can be validated and applied across multiple settings?
8. How can user experience evaluations be used to make judgments about the quality of different types of shared sanitation and of shared sanitation interventions?
9. What additional measures are needed to understand the impacts of different kinds of sanitation further down the service chain—for example, regarding pathogen exposures and/or quality of life of sanitation workers?^53^

## CONCLUSION

In many settings, private household sanitation is a distant prospect. So, understanding the conditions under which high-quality shared sanitation leads to positive user experiences, seeking to improve these conditions, and developing rigorous measures may lead to adequate prioritization of higher quality shared sanitation in dense urban areas. Therefore, we suggest a final research question to be added to the agenda:

10. How can we promote the prioritization of improving the quality of shared sanitation in these dense urban contexts?
